# Laboratory organisation and management of SARS-CoV-2 infection in Niger, West Africa

**DOI:** 10.4102/ajlm.v9i1.1308

**Published:** 2020-12-21

**Authors:** Abdourahamane Yacouba, Adamou Lagaré, Daouada Alhousseini Maiga, Halimatou Moumouni Sambo, Sani Ousmane, Zelika Hamidou Harouna, Boubacar Marou, Maman K. Sanoussi, Balki Aoula, Ali Amadou, Hassane Boureima, Saidou Amatagas, Abdoulaye Ousmane, Eric Adehossi, Saidou Mamadou

**Affiliations:** 1 Faculté des Sciences de la Santé, Université Abdou Moumouni, Niamey, Niger; 2 Laboratory Team COVID-19, Niamey, Niger; 3 Centre de Recherche Médicale et Sanitaire, Niamey, Niger; 4 Direction des Laboratoires de Santé, Ministère de la Santé Publique, Niamey, Niger; 5 Hôpital National Amirou Boubacar Diallo, Niamey, Niger; 6 Hôpital de l’Amitié Niger-Turquie, Niamey, Niger; 7 Hôpital Général de Référence de Maradi, Maradi, Niger; 8 Hôpital National de Zinder, Zinder, Niger; 9 Faculté des Sciences de la Santé, Université Dan Dicko Dankoulodo de Maradi, Maradi, Niger; 10 COVID-19 Experts Group, Niamey, Niger

**Keywords:** severe acute respiratory syndrome coronavirus-2, SARS-CoV-2, coronavirus disease 2019, COVID-19, laboratory diagnosis, West Africa, Niger

## Abstract

**Background:**

As the coronavirus disease 2019 (COVID-19) pandemic unfolds, laboratory services have been identified as key to its containment. This article outlines the laboratory organisation and management and control interventions in Niger.

**Intervention:**

The capitol city of Niger, Niamey, adopted a ‘National COVID-19 Emergency Preparedness and Response Plan’ to strengthen the preparedness of the country for the detection of severe acute respiratory syndrome coronavirus-2. Laboratory training and diagnostic capacity building were supported by existing active clinical and research laboratories for more rapid and practicable responses. The National Reference Laboratory for Respiratory Viruses located at the *Centre de Recherche Médicale et Sanitaire* was designated as the reference centre for COVID-19 testing. The national plan for COVID-19 testing is being gradually adopted in other regions of the country in response to the rapidly evolving COVID-19 emergency and to ensure a more rapid turn-around time.

**Lessons learnt:**

After the decentralisation of COVID-19 testing to other regions of the country, turn-around times were reduced from 48–72 h to 12–24 h. Reducing turn-around times allowed Niger to reduce the length of patients’ stays in hospitals and isolation facilities. Shortages in testing capacity must be anticipated and addressed. In an effort to reduce risk of shortages and increase availability of reagents and consumables, Niamey diversified real-time reverse transcriptase–polymerase chain reaction kits for severe acute respiratory syndrome coronavirus-2 detection.

**Recommendations:**

Continued investment in training programmes and laboratory strategy is needed in order to strengthen Niger’s laboratory capacity against the outbreak.

## Background

In December 2019, a new viral respiratory infection emerged. The infection was first detected and reported in the Chinese province of Hubei and has since spread globally, affecting people and socio-economic development in both developing and developed countries. The disease, later named the coronavirus disease 2019 (COVID-19) and declared a global pandemic by the World Health Organization, is caused by a species of coronavirus known as severe acute respiratory syndrome coronavirus-2 (SARS-CoV-2), which belongs to the family *Coronaviridae* and the genus *Betacoronavirus.*^1,2,3^ Worldwide, real-time reverse transcriptase–polymerase chain reaction (rRT-PCR) is the gold standard method used to detect the presence of SARS-CoV-2 RNA in clinical specimens.

As of 15 June 2020, 175 503 laboratory-confirmed COVID-19 cases were reported in Africa, with 4111 deaths.^4^ These figures make Africa the continent least affected by this pandemic, with the most significantly affected countries in the region being South Africa (70 038 cases), Nigeria (16 085 cases), Ghana (11 422 cases), and Algeria (10 919 cases).^4^

The index case in the Republic of Niger was detected on 19 March 2020. This was an imported case involving a 36-year old man who arrived by road from Burkina Faso. Since the official declaration of the index case, a total of 980 cases have been confirmed as of 15 June 2020.^5^

As this pandemic unfolds, laboratory services have been identified as key to containment efforts. This article outlines the laboratory organisation and management and control interventions in Niger.

## Description of the intervention

### Ethical consideration

This article followed all ethical standards for research without direct contact with human or animal subjects.

### Organisational response

Severe acute respiratory syndrome coronavirus-2 is an emerging respiratory pathogen spreading rapidly through the general population. Niamey, the capitol city of Niger, adopted the ‘National COVID-19 Emergency Preparedness and Response Plan’ (available from https://tinyurl.com/y2x3vpmt) to strengthen Niger’s preparedness for the detection of SARS-CoV-2. This plan details the procedures for containment, contact tracing and screening based on the risk of spread. The plan is structured around five major strategic axes including reinforcement of coordination, strengthening of epidemiological surveillance, strengthening of health services capacities, reinforcement of risk communication and community engagement, and, lastly, the creation of isolation facilities.

Eight committees have been set up to implement the National COVID-19 Emergency Preparedness and Response Plan, one of which is the Laboratory and Research Team. The National COVID-19 Emergency Preparedness and Response Plan provides $16 484 884.48 (United States dollars) to enable expedited building and implementation of SARS-CoV-2 testing capacity in the eight regions of the country in tune with the expansion of the pandemic. The Laboratory and Research Team, which is charged with the responsibility of improving laboratory capacity and capability, is structured into three groups comprising a pre-analytical group (sample collection, inactivation, identification numbers), an analytical group (rRT-PCR testing) and a post-analytical group (validation and reporting test results).

For more rapid and practicable responses, laboratory diagnostic capacity building is being supported by existing active clinical and research laboratories. The National Reference Laboratory for Respiratory Viruses located at the *Centre de Recherche Médicale et Sanitaire* (CERMES) was designated as the reference centre for COVID-19 testing. The Veterinary Research Laboratory ‘*Laboratoire Central de l’Elevage’*, the National Reference Laboratory for HIV and Tuberculosis, the National Hospital of Niamey and the Research Institute for Sustainable Development supported the laboratory response by providing equipment for PCR techniques. Moreover, seven laboratory technicians from these centres, who have had extensive training on PCR techniques, were mobilised by the Ministry of Health, in addition to the CERMES technicians, to support the response by performing the rRT-PCR testing at CERMES, which received and analysed samples collected from across the country. As part of COVID-19 response, Niamey and its partners provided logistical air support for sample transport from remote regions such as Diffa, Zinder, Maradi, Tahoua and Agadez.

**FIGURE 1:**
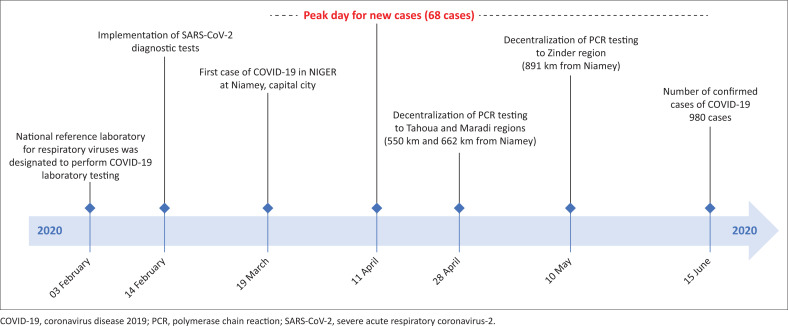
Laboratory organisation and adaptation for COVID-19 pandemic in Niger. The 68 cases (in red) recorded on 11 April 2020 denote the peak between 19 March 2020 (index case) and 15 June 2020.

Gradually, the national plan on COVID-19 testing is being adopted in other regions of the country in response to the rapidly evolving COVID-19 emergency and to ensure a more rapid turn-around time (TAT). This adoption involves gradual decentralisation of the SARS-CoV-2 RNA rRT-PCR assays to three regions, Tahoua, Maradi and Zinder, located 550, 662 and 891 kilometres from Niamey (Figure 1). The choice of these regions was based on several factors, including the distance from the capital city, the number of close contacts in these regions, the number of confirmed cases at the time of the decentralisation and the capacity of the laboratory facilities on site.

The Tahoua laboratory analysed samples collected from the Tahoua and Agadez regions. Samples collected from the Zinder and Diffa regions were analysed in Zinder, whereas the Maradi laboratory analysed samples collected in the Maradi region. Samples collected from the Niamey, Dosso and Tillabery regions were analysed at CERMES. Logistics and testing capacity are handled by CERMES, which reported the availability of the reagents and materials needed to sample patients and to perform the rRT-PCR on a weekly basis to the Ministry of Health.

### Laboratory diagnosis

Severe acute respiratory syndrome coronavirus-2 is classified as a Risk Group 3 human pathogen, similar to Middle East respiratory syndrome coronavirus and severe acute respiratory syndrome coronavirus.^6^ The interim guidance from the World Health Organization suggests that non-propagative processing and handling of specimens containing SARS-CoV-2 must be performed in standard biosafety level-2 laboratories.^7,8^ Consequently, Niamey leveraged the safe practices and techniques, safety equipment and appropriate facility design of CERMES to reduce or eliminate exposure of laboratory workers to SARS-CoV-2-containing materials according to the World Health Organization requirements.^7^ Before the decentralisation of SARS-CoV-2 RNA rRT-PCR assays to other regions, samples collected from across the country were sent to CERMES. Samples from remote regions such as Diffa, Zinder and Agadez were transported using flights in accordance with category B transportation regulations.^9^ In an effort to help close contact management, sample collection was performed during home visits.

Ribonucleic acid extraction from samples was performed manually using QIAamp^®^ Viral RNA Mini Kit (250) (QIAGEN GmbH, Hilden, Germany) or Nucleic Acid Isolation or Purification Reagent (DaAn Gene Co., Ltd, Guangzhou, China) according to the manufacturer’s instructions. Two teams of four laboratory technicians were dedicated to RNA extraction at CERMES. These teams were often overworked due to the large volume of samples handled.

Qualitative rRT-PCR assays were performed using the nucleic acid testing kit (DaAn Gene Co., Ltd, Guangzhou, China).^10^ Two target genes, the open reading frame1ab (*ORF1ab*) and nucleocapsid protein (*N*), were simultaneously amplified. A cycle threshold value lower than 40 was defined as a positive test result, and a cycle threshold value of 40 or more was defined as a negative outcome according to the manufacturer’s protocol. Alternatively, rRT-PCR targeting the *RdRp* gene of the SARS-CoV-2 or SARS-like coronavirus was performed using the LightMix^®^ Modular SARS-CoV-2 *RdRp* (TIB Molbiol, Berlin, Germany). Positive samples for *RdRp* gene were confirmed by performance of rRT-PCR for the detection of the *E* gene using LightMix^®^ SarbecoV *E* gene plus equine arteritis virus control (TIB Molbiol, Berlin, Germany). Given the extraordinary demand for reagents and consumables, risk of supply shortages became the main issue. Niamey experienced a shortage of RNA extraction kits and this led to the prioritisation of the testing of vulnerable people, health professionals and patients requiring hospitalisation.

**FIGURE 2:**
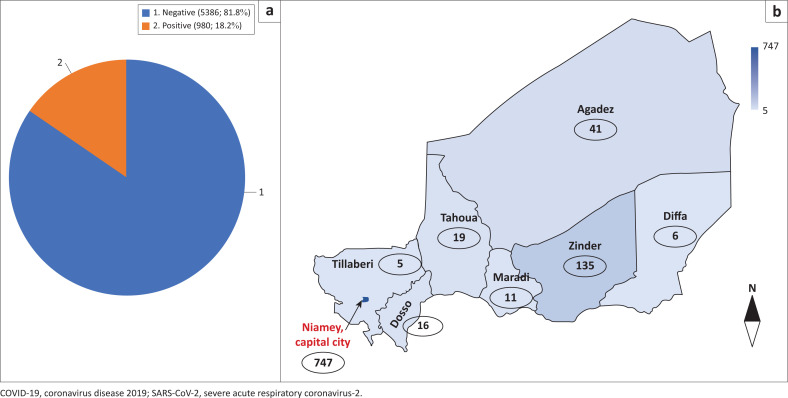
COVID-19 situation in Niger as of 15 June 2020. (a) Numbers of SARS-CoV-2 RNA-positive patients. (b) Map of Niger showing the distribution of SARS-CoV-2-positive patients according to region.

As of 15 June 2020, a total of 5386 samples had been tested for SARS-CoV-2 in Niger. Of these, 980 (18.2%) were confirmed positive (Figure 2). All test results, positive or negative, were immediately reported to the national committee, which is responsible for communication on the pandemic. The highest number of new daily confirmed cases (68 cases) was detected on 11 April 2020.

In March and April 2020, before the decentralisation of SARS-CoV-2 RNA rRT-PCR assays to other regions, the number of samples received exceeded the capacity of the single centralised laboratory earmarked for the testing. Consequently, TATs were between 48 h and 72 h. This TAT length often created a disconnect between clinicians and laboratory staff. Indeed, according to the clinicians, the TAT of 48–72 h delayed treatment and increased patients’ length of stay, particularly in isolation facilities where patients are often asymptomatic. After decentralisation, on 15 June, 2020, the TAT had decreased significantly to 12 h at Maradi and between 12 h and 24 h at Tahoua, Zinder and CERMES, Niamey.

## Lessons learnt

Experience gained in the management of previous outbreaks (Rift Valley Fever and meningitis) helped Niamey to build a quick response to the COVID-19 pandemic. However, additional control efforts are needed to improve the laboratory strategy and response against COVID-19 in the country. Firstly, a well-coordinated laboratory strategy and operational plan is needed in order to address the shortcomings of the existing plan. Moreover, considering the importance of improved SARS-CoV-2 laboratory capacity, the country should provide extensive training to laboratory technicians in preparation for rapid expansion of laboratory diagnostic capacity. Secondly, laboratory TAT is critical in determining the success of both the laboratory response programme and the management of patients. Reducing the TAT allowed reductions in patients’ length of stay in hospitals and isolation facilities. Thirdly, communication between clinical and laboratory staff needs to be improved in order to ensure that laboratory results are understandable to clinicians.

Fourthly, the shortages in testing capacity need to be anticipated and addressed. If capacity is exceeded, priority should be given to the testing of vulnerable patients, health professionals and patients requiring hospitalisation. Niamey diversified rRT-PCR kits for SARS-CoV-2 detection to reduce the risk of shortages and increase the availability of reagents. It has been demonstrated that RNA extraction kits from different manufacturers are interchangeable.^11^

However, diversifying the type of rRT-PCR kits for SARS-CoV-2 detection can lead to some variation in the detection rate between kits.^12^ It has been well documented that the limit of detection of different rRT-PCR kits can differ substantially.^13,14^ Therefore, care must be taken in the interpretation of the results when using different kits for the monitoring of patients. Fifthly, the overwhelming number of specimens to test highlighted the need for fast methods to extract viral RNA. Ribonucleic acid extraction from samples was performed manually in Niger. In addition to the high risk of contamination, manual extraction of viral RNA is time consuming and requires a large number of laboratory professionals.

Lastly, amplification of multiple target genes (e.g. *N, E, RpRd* genes) could be used to avoid invalid results and increase sensitivity for detection of new genomic variants.^15,16^

## Recommendations

Despite TAT improvement in the laboratory management of COVID-19 testing, several additional control efforts are needed to improve the laboratory response against COVID-19 in Niger. Considering the importance of improved SARS-CoV-2 laboratory capacity, continued investment in training programmes and laboratory strategy is needed to systematically guide the laboratory response against the outbreak.

## References

[1] BBC News Coronavirus disease named Covid-19 [homepage on the Internet]. [cited 2020 May 31]. Available from: https://www.bbc.com/news/world-asia-china-51466362

[2] WHO Director-General’s opening remarks at the media briefing on COVID-19 – 25 May 2020 [homepage on the Internet]. [cited 2020 May 31]. Available from: https://www.who.int/dg/speeches/detail/who-director-general-s-opening-remarks-at-the-media-briefing-on-covid-19---25-may-2020

[3] Gorbalenya AE, Baker SC, Baric RS, et al The species severe acute respiratory syndrome-related coronavirus: Classifying 2019-nCoV and naming it SARS-CoV-2. Nat Microbiol. 2020;5(5):536–544. 10.1038/s41564-020-0695-z 32123347 PMC7095448

[4] World Health Organisation Coronavirus disease (COVID-19) situation reports [homepage on the Internet]. [cited 2020 Jun 16]. Available from: https://www.who.int/emergencies/diseases/novel-coronavirus-2019/situation-reports

[5] Coronavirus: Le Niger enregistre son premier cas sikafinance.com [homepage on the Internet]. [cited 2020 Aug 09]. Available from: https://www.sikafinance.com/marches/coronavirus-le-niger-enregistre-son-premier-cas_21336

[6] Iwen PC, Stiles KL, Pentella MA Safety considerations in the laboratory testing of specimens suspected or known to contain the Severe Acute Respiratory Syndrome Coronavirus 2 (SARS-CoV-2). Am J Clin Pathol. 2020 4 15;153(5):567–570. 10.1093/ajcp/aqaa047 32190890 PMC7184496

[7] World Health Organisation Laboratory testing for coronavirus disease 2019 (COVID-19) in suspected human cases: Interim guidance, 2 March 2020 [homepage on the Internet]. World Health Organization; 2020 [cited 2020 Sep 05]. Available from: https://www.WHO/COVID-19/laboratory/2020.4

[8] World Health Organisation Laboratory biosafety guidance related to the novel coronavirus (2019-nCoV) [homepage on the Internet]. World Health Organization; 2020 [cited 2020 Sep 18]. Available from: https://www.who.int/docs/default-source/coronaviruse/laboratory-biosafety-novel-coronavirus-version-1-1.pdf?sfvrsn=912a9847_2.%20Accessed%20March%207,%202020

[9] World Health Organisation Guidance on regulations for the transport of infectious substances [homepage on the Internet]. [cited 2020 Sep 05]. Available from: https://www.who.int/csr/resources/publications/biosafety/WHO_CDS_CSR_LYO_2005_22r%20.pdf?ua=1

[10] World Health Organisation WHO emergency use listing for in vitro diagnostics (IVDs) detecting SARS-CoV-2 nucleic acid [homepage on the Internet]. [cited 2020 Sep 18]. Available from: https://www.who.int/diagnostics_laboratory/200710_eul_sars_cov2_product_list.pdf?ua=1

[11] Lim KL, Johari NA, Wong ST, et al A novel strategy for community screening of SARS-CoV-2 (COVID-19): Sample pooling method. PLoS One. 2020;15(8):e0238417 10.1371/journal.pone.0238417 32857823 PMC7454965

[12] Van Kasteren PB, Van der Veer B, Van den Brink S, et al Comparison of seven commercial RT-PCR diagnostic kits for COVID-19. J Clin Virol. 2020 5 8;128:104412 10.1016/j.jcv.2020.104412 32416600 PMC7206434

[13] Hogan CA, Sahoo MK, Huang C, et al Comparison of the panther fusion and a laboratory-developed test targeting the envelope gene for detection of SARS-CoV-2. J Clin Virol. 2020 6 1;127:104383 10.1016/j.jcv.2020.104383 32353760 PMC7195328

[14] Wang X, Yao H, Xu X, et al Limits of detection of 6 approved RT–PCR kits for the novel SARS-coronavirus-2 (SARS-CoV-2). Clin Chem. 2020 7 1;66(7):977–979. 10.1093/clinchem/hvaa099 32282874 PMC7184447

[15] Tahamtan A, Ardebili A Real-time RT-PCR in COVID-19 detection: Issues affecting the results. Expert Rev Mol Diagn. 2020 5 3;20(5):453–454. 10.1080/14737159.2020.1757437 32297805 PMC7189409

[16] Penarrubia AL, Ruiz M, Porco R, et al Multiple assays in a real-time RT-PCR SARS-CoV-2 panel can mitigate the risk of loss of sensitivity by new genomic variants during the COVID-19 outbreak. Int J Infect Dis. 2020;97:225–229. 10.1016/j.ijid.2020.06.027 32535302 PMC7289722

